# Polymorphic variants of the hOGG1, APEX1, XPD, SOD2, and CAT genes involved in DNA repair processes and antioxidant defense and their association with breast cancer risk

**DOI:** 10.18699/vjgb-24-48

**Published:** 2024-07

**Authors:** А.А. Timofeeva, V.I. Minina, A.V. Torgunakova, О.А. Soboleva, R.А. Тitov, Ya.А. Zakharova, M.L. Bakanova, А.N. Glushkov

**Affiliations:** Federal Research Center of Coal and Coal Chemistry of the Siberian Branch of the Russian Academy of Sciences, Kemerovo, Russia; Federal Research Center of Coal and Coal Chemistry of the Siberian Branch of the Russian Academy of Sciences, Kemerovo, Russia Kemerovo State University, Kemerovo, Russia; Federal Research Center of Coal and Coal Chemistry of the Siberian Branch of the Russian Academy of Sciences, Kemerovo, Russia Kemerovo State University, Kemerovo, Russia; Federal Research Center of Coal and Coal Chemistry of the Siberian Branch of the Russian Academy of Sciences, Kemerovo, Russia; Federal Research Center of Coal and Coal Chemistry of the Siberian Branch of the Russian Academy of Sciences, Kemerovo, Russia Kemerovo State University, Kemerovo, Russia; Federal Research Center of Coal and Coal Chemistry of the Siberian Branch of the Russian Academy of Sciences, Kemerovo, Russia Kemerovo State University, Kemerovo, Russia; Federal Research Center of Coal and Coal Chemistry of the Siberian Branch of the Russian Academy of Sciences, Kemerovo, Russia; Federal Research Center of Coal and Coal Chemistry of the Siberian Branch of the Russian Academy of Sciences, Kemerovo, Russia

**Keywords:** breast cancer, luminal B subtype, hOGG1, APEX1, XPD, SOD2, CAT, рак молочной железы, люминальный подтип В, hOGG1, APEX1, XPD, SOD2, CAT

## Abstract

Breast cancer is one of the leading causes of mortality among women. The most frequently encountered tumors are luminal tumors. Associations of polymorphisms in the hOGG1 (rs1052133), APEX1 (rs1130409), XPD (rs13181), SOD2 (rs4880), and CAT (rs1001179) genes were studied in 313 nonsmoking postmenopausal patients with luminal B subtype breast cancer. The control group consisted of 233 healthy nonsmoking postmenopausal women. Statistically significant associations of the XPD and APEX1 gene polymorphisms with the risk of developing luminal B Her2-negative subtype of breast cancer were observed in a log-additive inheritance model, while the CAT gene polymorphism showed an association in a dominant inheritance model (OR = 1.41; CI 95 %: 1.08–1.85; Padj.= 0.011; OR = 1.39; CI 95 %: 1.07–1.81; Padj = 0.013 и OR = 1.70; CI 95 %: 1.19–2.43; Padj = 0.004, respectively). In the group of elderly women (aged 60–74 years), an association of the CAT gene polymorphism with the risk of developing luminal B subtype of breast cancer was found in a log-additive inheritance model (OR = 1.87; 95 % CI: 1.22–2.85; Padj = 0.0024). Using MDR analysis, the most optimal statistically significant 3-locus model of gene-gene interactions in the development of luminal B Her2-negative subtype breast cancer was found. MDR analysis also showed a close interaction and mutual enhancement of effects between the APEX1 and SOD2 loci and the independence of the effects of these loci from the CAT locus in the formation of luminal B subtype breast cancer.

## Abstract (ru)

Онкологические заболевания молочной железы – одна из ведущих причин смертности у женщин. Рак молочной железы относится к числу распространенных мультифакториальных полигенных заболеваний, реализующихся в результате сочетанного взаимодействия генетических и средовых факторов. Наиболее часто встречаются люминальные опухоли. Люминальный подтип В рака молочной железы характеризуется худшим прогнозом и ранними рецидивами. Для изучения генетических факторов риска развития злокачественных новообразований молочной железы необходимо определить полиморфные варианты генов, играющих важную роль в канцерогенезе, к числу которых относятся гены репарации ДНК и системы антиоксидантной защиты. Изучены ассоциации полиморфизмов генов hOGG1 (rs1052133), APEX1 (rs1130409), XPD (rs13181), SOD2 (rs4880) и CAT (rs1001179) у 313 некурящих пациенток в постменопаузе с диагнозом люминального подтипа В Her2-негативного рака молочной железы. В контрольную группу вошли 233 здоровые некурящие женщины в постменопаузе. Зарегистрированы с поправкой на возраст статистически значимые ассоциации полиморфных вариантов генов XPD (rs13181) и APEX1 (rs1130409) с риском развития люминального подтипа В Her2-негативного рака молочной железы в лог-аддитивной модели наследования, гена CAT (rs1001179) – в доминантной модели OR = 1.41; CI 95 %: 1.08–1.85; Padj = 0.011; OR = 1.39; CI 95 %: 1.07–1.81; Padj = 0.013 и OR = 1.70; CI 95 %: 1.19–2.43; Padj = 0.004 соответственно). В группе женщин пожилого возраста (60–74 года) выявлена ассоциация вариантов гена CAT (rs1001179) с риском развития рака молочной железы в лог-аддитивной модели наследования (OR = 1.87; CI 95 %: 1.22–2.85; Padj = 0.0024). С помощью MDR-анализа найдена наиболее оптимальная статистически значимая 3-локусная модель межгенных взаимодействий при развитии онкозаболеваний молочной железы люминального подтипа В. MDR-анализ показал также тесное взаимодействие и взаимное усиление эффектов между локусами APEX1 и SOD2 и независимость эффектов данных локусов от эффекта локуса САТ при формировании люминального подтипа В рака молочной железы

## Introduction

Malignant transformations of the breast are the most wide spread oncological pathologies, by amount of deaths they take second place in world statistics (Siegel, 2021). Age, excess weight, heritage can be referred to as risk factors for oncopathology of breasts. Genetical, reproductive and hormonal factors can make a significant contribution to breast cancer. According to literature data, hormonal (luminal) malignancies are the most widespread (Ignatiadis, Sotiriou, 2013). Luminal B subtype of breast cancer, as opposed to luminal A subtype, is characterized by poor prognosis, early recurrence and high frequency of metastases in lymph nodes (Nishimura et al., 2010).

Breast cancer (BC) is a complex disorder with a high level of heterogeneity. The most well-studied markers of hereditary risk of BC are mutations in genes like BRCA1/2, PALB2, TP53. They influence the risk increase for BC more than twofold in comparison with the whole population. BC that is linked with germinal mutations in BRCA1 has a triple negative phenotype (70–85 %), while ER-positive cases can be detected in carriers of mutations in the BRCA2, ATM, CHEK2 and PALB2 genes (Breast Cancer Association Consortium, 2021).

Meanwhile the majority of BC cases are sporadic (only from 5 to 10 % cases of BC are hereditary forms). There is a need for significant prognostic markers for sporadic forms of BC that can allow us to determine the group of risk to decrease mortality and morbidity.

Genome-wide association studies (GWASs) allowed to register over 170 loci of susceptibility for malignant breast transformation development, among them the biggest contribution can be made by single nucleotide polymorphisms (Michailidou et al., 2017; Ferreira et al., 2019). In Caucasian women, via GWAS, 32 loci associated with BC risk were identified. Five loci showed associations (P < 0.05) in the opposite direction between luminal and non-luminal subtypes of BC. In silico studies demonstrated that these five loci consist of cell-specific enhancers that differ in normal, luminal and basal cells of breasts (Zhang H. et al., 2020). A large number of variants detected by similar studies as a rule are located in regulatory non-coding regions, especially in distal enhancers and transcription factor binding sites (Pan et al., 2021).

Variants of DNA repair genes among different biomarkers are of greatest interest. DNA aberrations such as oxidative and reductive nitrogen bases, adducts and mutations induced by methylation agents can be recovered by enzymes of base excision repair (BER).

The hOGG1 gene encodes a key enzyme of the BER pathway, bifunctional DNA-glycosilase/β-lyase, which excludes residues of 8-oxoguanine. The most well-studied and useful hOGG1 polymorphic variant is rs1052133, which causes substitution of serine with cysteine in region 326 of the protein, decreasing the ability for repair activity (Niu et al., 2012). In a study using a BC cell line (HCC1937), it was shown that these cells are able to accumulate high levels of 8-oxoguanine in comparison with to normal glandular tissue (Nyaga et al., 2006).

Another gene of the BER pathway is APEX1, which encodes apurinic/apirimidinic endonuclease that can delete DNA sites with no nitrogen bases. APEX1 rs1130409 polymorphic variant is linked to transversion of thymine to guanine in the 5th exon and causes substitution of asparagine acid with glutamine acid (Asp148Glu). It is associated with the ability of this enzyme to interact with other components of BER, thus decreasing the effectiveness of repair (Hadi et al., 2000).

Nucleotide excision repair (NER) plays a crucial role in stabilization of genome structure due to its ability to recover a high spectrum of DNA mutations (Sugasawa, 2010). One of the key components of this pathway is the XРD gene that encodes helicase, which participates in DNA unwinding and recognition of adducts and thymine dimers (Fontana et al., 2008). Substitution of adenine with cytosine in region 2251 of the gene (rs13181) promotes replacement of lysine by glutamine in region 751 of the protein, thus changing its configuration and causing interaction with helicase activator (Romaniuk et al., 2014).

Oxidative stress is one the most important factors in cancerogenesis caused by active forms of oxygen production that can affect DNA and initiate lipid peroxidation and modification of protein molecules (Caporaso, 2003; Tas et al., 2005). Effectiveness of autoxidation system performance is ensured by individual genetic properties. Catalase (CAT) and superoxide dismutase (SOD2) refer to proteins that can protect cells against oxidative stress (Ambrosone, 2000).

CAT is a key enzyme involved in neutralization of active oxygen forms via breakdown of hydrogen peroxide to water and oxygen (Ambrosone, 2000). Allele variants of this gene are associated with reduction of catalytic activity of this enzyme. rs1001179 is a well-studied polymorphic variant in the promoter region of the gene that can influence gene expression and cause a decrease in enzyme activity (Forsberg et al., 2001; Bastaki et al., 2006). A hypothesis about a link between estrogen exposure and catalase activity was made. It was shown that exposition of normal epithelial cells of human breasts to estradiol decreases the activity of cellular catalase (Forsberg et al., 2001).

Manganese-dependent superoxide dismutase works in the antioxidative system and is expressed in mitochondria. Transition of cytosine to thymine in the 47th region of the gene (rs4880) causes alanine-to-valine substitution in the 16th region of the protein and alteration of the secondary structure of the signal peptide. Destabilization of its alpha-helix domain decreases import of the protein from the cytoplasm to the mitochondria matrix causing enzyme absence. For T variant, mRNA instability is typical (Sutton et al., 2005). Association of this single nucleotide polymorphism with SOD2 overexpression and accumulation of genotoxic oxygen peroxide has already been described (Ji et al., 2012).

Based on the above, the aim of this study was the analysis of association of loci hOGG1 (rs1052133), APEX1 (rs1130409), XPD (rs13181), SOD2 (rs4880) and CAT (rs1001179) with BC development risk in women with luminal B Her2-negative subtype

## Materials and methods

Overall, 2,150 women with breast cancer that are Kemerovo region residents were observed. Inclusion criteria of patients in the study were as follows: Caucasian, female, age over 40, postmenopausal, previously diagnosed with luminal B Her2- negative BC, absence of family forms of oncopathology. Exclusion criteria were: smoking, oncopathology forms in anamnesis, relatives with oncopathology.

313 non-smoking women were selected from the whole sample of patients (median age 60.88 ± 0.35), 42.04 % had the I stage of disease, 42.04 % had the II stage, 13.38 and 2.55 % patients were diagnosed with the III and IV stages of BC, respectively. Metastases in lymph nodes and/or in distal organs were observed in 51 women. All patients were observed by medicals of Kuzbass Clinical Oncological Dispensary using a whole complex of diagnostics methods, after that it became possible to make a certain pathomorphological diagnosis for each woman. Classification of subtypes was based on expressional parameters of estrogen (ER) and progesterone (PR) receptors and also those of receptor tyrosine kinase (Her2) and level of proliferative activity of Ki-67 (Gold-hirsch et al., 2013).

233 Kemerovo region residents were included into the control group without any symptoms of oncological disorders (median age 58.44 ± 0.34). Inclusion criteria in the control group were: Caucasian, female, age over 40 years, postmenopausal. Exclusion criteria were: smoking, oncological cases in anamnesis, relatives with oncopathology. Age characteristics of the observed groups (according to the WHO recommendations of 2016) are presented in Table 1.

**Table 1.**
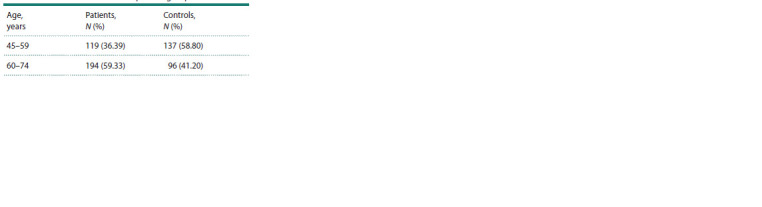
Characteristics of the comparison groups

This study was approved by the ethics committee of the Federal Research Center of Coal and Coal Chemistry of SB RAS according to the statements of the Helsinki declaration (ratified in 2000). Collection of data and samples of peripheral blood was conducted after receiving voluntary informed consent from patients and healthy individuals.

DNA was purified from peripheral blood via the standard method of phenol-chloroform extraction (Sambrook et al., 1989). Variants of the hOGG1 (rs1052133), APEX1 (rs1130409), XPD (rs13181), and CAT (rs1001179), SOD2 (rs4880) genes were genotyped by real-time PCR using Taq- Man primers from SibDNA kits (SibDNA, Novosibirsk, Russia). Amplification and detection of the results were performed using the CFX96 amplificator (BioRad, USA).

SNPStats (http://bioinfo.iconcologia.net/SNPstats) and STATISTICA 10.0 (StatSoft Inc., Tulsa, Оklahoma, USA) programs were used for statistical processing of the obtained results. Analysis of rare allele frequency, accordance to Hardy– Weinberg equilibrium were provided by available online sources (https://gene-calc.pl/hardy-weinberg-page and http:// www.quantpsy.org/chisq/chisq.htm, respectively). Statistically significant results were accepted with p < 0.05. For minimization of type I statistical error, multiple comparisons problem was used. Using age parameter, we performed a logistic regression analysis with odds ratio (OR) calculation (with 95 % confidence interval). The most convenient statistical model with the lowest value was selected using Akaike Information Criteria (AIC). With Multifactor Dimensionality Reduction (MDR), which allows to evaluate all possible models of SNP combinations, we investigated intergenic interactions. Contribution of each gene and/or their interactions were evaluated by H-parameter (caused by entropy) and represented as a percentage (%) (Moore et al., 2006). To perform this analysis, the program package of MDR 3.2.0 was used (Computational Genetics Laboratory, Philadelphia, Pennsylvania, USA).

## Results

Investigation of the hOGG1, APEX1, XPD, SOD2 and CAT genes polymorphic variants was conducted in cohorts of nonsmoking women with luminal B subtype of BC and healthy women of similar age (Table 2).

**Table 2.**
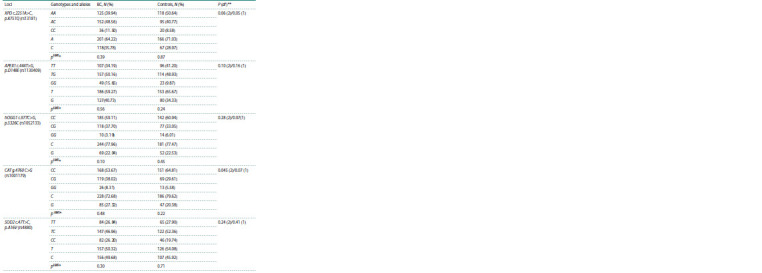
Distribution of DNA repair and antioxidant system genes polymorphic variants in the study groups * Accordance to Hardy–Weinberg equilibrium (HWE); ** level of significance after comparison of alleles and genotypes frequency in the study groups.

Distribution of alleles and genotypes in the studied groups corresponds to Hardy–Weinberg equilibrium and to parameters observed in Caucasian population (http://www.ensembl. org/Homo_sapiens). No statically significant differences were detected between different groups of patients (malignancy stage, its localization, metastases development). Significant differences between genotypes and alleles distribution in DNA repair and antioxidant system genes, taking into account the Bonferroni correction were not detected in study groups

Analysis of different hereditary models with correction for age allowed to detect association between the risk of luminal B Her2-negative BC development and XPD (rs13181) and APEX1 (rs1130409) in the log-additive model, and CAT (rs1001179) in the dominant model (OR = 1.41; CI 95 %: 1.08–1.85; Padj = 0.011; OR = 1.39; CI 95 %: 1.07–1.81; Padj = 0.013 and OR = 1.70; CI 95 %: 1.19–2.43; Padj = 0.004 respectively).

Distribution of genotypes and alleles of the studied genes in different age groups of patients with BC and healthy women is presented in Table 3.

**Table 3.**
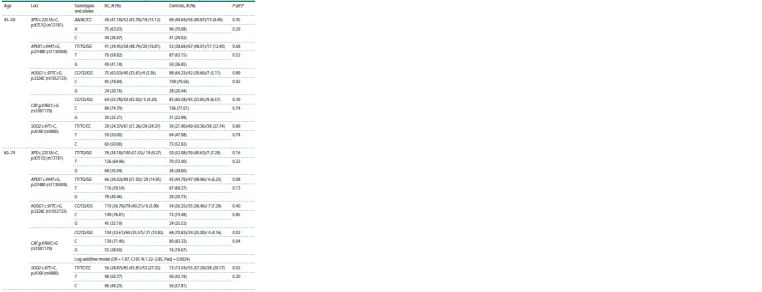
Distribution of different variants of DNA repair and antioxidative system genes in different study groups * Level of significance in comparison of alleles and genotypes distribution between different study groups.

Analysis of different hereditary models allowed to reveal links between polymorphic variants of the CAT (rs1001179) gene with the risk of luminal B Her2-negative BC development in elder patients (60–74 years) in the log-additive model (OR = 1.87; CI 95 %: 1.22–2,85; Padj = 0.0024).

Via the MDR method, the most optimal 3-loci model of intergenic interactions with a high level of precision, minimal rate of error for BC risk prediction and maximal level of reproducibility evaluation was obtained (Table 4).

**Table 4.**
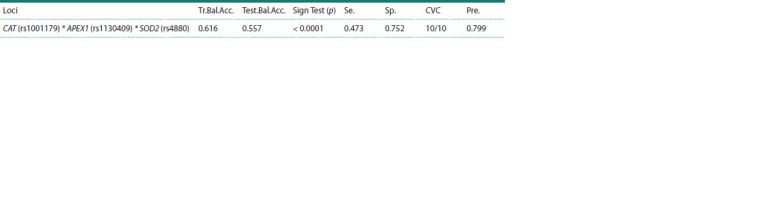
Significant intergenic interactions during BC development Note. Tr.Bal.Acc. – training balanced accuracy; Test.Bal.Acc. – testing balanced accuracy; Sign Test (p) – test for significance; Se. – sensitivity; Sp. – specificity; CVС – repeatability of the result; Pre. – precision of the model

**Fig. 1.**
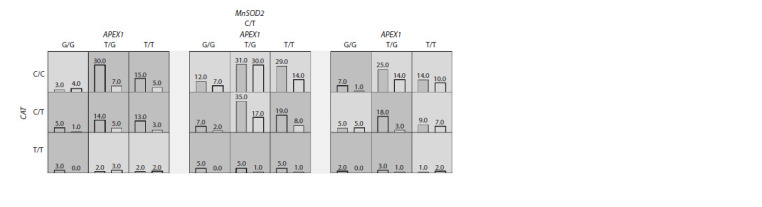
Combination of genotypes for the 3-loci model of САТ (rs1001179), APEX1 (rs1130409) and SOD2 (rs4880) that can predispose to the risk of luminal B Her2-negative BC development Dark grey cells – genotypes of increased risk, light grey cells – genotypes of decreased risk (left columns in the cells – patients with BC, right columns – healthy women).

Analysis of the model in contingency tables, which represent all possible variants for the 3-loci model, revealed 12 protective and 15 risk combinations for luminal B Her2- negative BC development (Fig. 1).

**Fig. 2.**
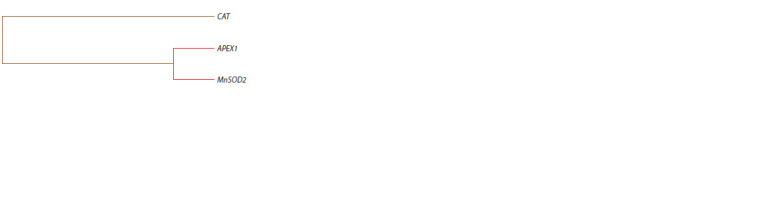
Dendrogram of intergenic interactions during formation of luminal B Her2-negative BC. Red – synergy of effects, brown – independent interaction.

The MDR analysis showed a simultaneous strengthening of effects between two loci, APEX1 (rs1130409) (H = 0.07 %) and SOD2 (rs4880) (H = 0.55 %), and also independence of their effects from САТ (rs1001179) (H = 0.44 %) during formation of luminal B Her2-negative BC (Fig. 2).

## Discussion

Sensitivity of an organism to air pollutants depends on the correct work of many enzyme systems, which include DNA repair and the antioxidative system. The level of breast tissues exposition to exo- and endogenous estrogens (providing DNA adducts formation) makes a big contribution to disease pathogenesis (Martucci, Fishman, 1993; Hanawalt, 2002). Estrogens are involved in regulation of antioxidative enzymes and can initiate oxidative mutations in DNA due to formation of active forms of oxygen during metabolic reactions (Tjønneland et al., 2004; Bergman et al., 2005; Silva et al., 2006; Liou, Storz,2010).

In one of the articles, influence of obesity on BC risk in female carriers of at least one minor allele of myeloperoxidase gene or DNA repair genes like GMT, MSH2, XPG and XRCC1 was detected (McCullough et al., 2015). In another study, it was shown that genes involved in oxidative stress and DNA repair can increase survival of women affected by breast oncopathologies (Rodrigues et al., 2012). At the same time there were no scientific works aimed at synergetic influence of DNA excision repair genes with genes of the antioxidative system on BC risk

DNA aberrations that are formed due to active forms of oxygen can be recovered via the BER and NER pathways. Results obtained in our work concerning APEX1 (rs1130409) association with BC risk are consistent with literature data (Mitra et al., 2008; Smith et al., 2008; Kim et al., 2013). Additionally, a link between the 444T allele and estrogen-positive BC development was revealed in Chinese women (Wang T. et al., 2018). Besides the repair function, this enzyme can perform oxidative-reductive activity of transcriptional factors (Kelley et al., 2012; Wang Z. et al., 2014). Redox activity of the protein contributes to synergy between the APEX1 (rs1130409) and SOD2 47 (rs4880) loci during BC formation.

The hOGG1 gene is another key component of the BER pathway. In our study, no links were found between hOGG1 (rs1052133) and BC risk. Similar results were demonstrated in the meta-analysis by M. Kamali et al. (2017), where association of 977G with BC wasn’t revealed in Caucasian as well as in Asian women (Kamali et al., 2017). At the same time, in a scientific work performed among Polish patients, hOGG1 977GG genotype contributed to the risk of BC development (Romanowicz et al., 2017).

Results of scientific studies that are aimed at XPD (rs13181) association with oncological disorders of the breast are not obvious. In works that were conducted using material of Canadian, Brazilian and Chinese women no significant results were obtained (Dufloth et al., 2005; Zhang L. et al., 2005; Onay et al., 2006). Observation of Indian patients allowed to reveal association of the 2251C allele with enhanced risk of BC (Samson et al., 2011). Later, a meta-analysis was conducted that showed an increased risk of BC in 2251C allele carriers in Caucasian and mixed populations (Yan et al., 2014). Similar results were demonstrated using Polish patients (Smolarz et al., 2019).

Manganese-dependent superoxide dismutase is one of the most important enzymes of the antioxidative system. Besides its own essential function (antioxidative activity), SOD2 protein has binding sites with different factors of transcription that are useful for its activation and are also involved in defense of cells against oxidative stress (Alateyah et al., 2022). Results of molecular and genetical studies of SOD2 (rs4880) association with BC risk are quite controversial. In our study, no influence of this polymorphic variant on the risk of malignant transformation development in breasts was detected. Similar results were obtained in the works conducted among Polish and Greek women (Jablonska et al., 2015; Kakkoura et al., 2016). In Mexican female patients, an association between the 47T allele of the SOD2 gene and luminal A subtype formation was detected, but not with luminal B (Gallegos-Arreola et al., 2022). In Iraqi and Taiwan, an association between this allele and increased BC risk was also detected (Tsai et al., 2012; Jabir, Hoidy, 2018).

Results of studies aimed to link the CAT (rs1001179) gene polymorphism with BC risk are still controversial. In some scientific works among American patients, an association between a decreased risk of BC and the -262 СС genotype was revealed in comparison with T allele carriers (Ahn et al., 2004, 2005). In our study, we got similar results. Ambiguous data were obtained by Y. Li et al. (2009), who registered a small decrease in BC risk in postmenopausal women with the CAT -262 CC genotype that were consumed a huge number of fruits and vegetables (over two portions a day). Among women with a small rate of fruits and vegetables consumption, CAT -262 CC was linked with an increased risk of BC (Li et al., 2009).

## Conclusion

The combined influence of DNA repair and antioxidative system genes variants on breast cancer risk was demonstrated. This work was conducted using material of postmenopausal women; to better understand the influence of individual genetical features on breast cancer development, it is also advisable to include younger women in experimental study.

To clarify the ability of the system of risk prognosis for BC risk evaluation, it is necessary to increase the number of studied patients to perform an additional study.

## Conflict of interest

The authors declare no conflict of interest.
